# The Era of Janus Kinase Inhibitors for Inflammatory Bowel Disease Treatment

**DOI:** 10.3390/ijms222111322

**Published:** 2021-10-20

**Authors:** Jin-Woo Kim, Su-Young Kim

**Affiliations:** Division of Gastroenterology, Department of Internal Medicine, Yonsei University Wonju College of Medicine, 20 Ilsan-ro, Wonju 26426, Korea; bluebloood@naver.com

**Keywords:** janus kinase inhibitor, inflammatory bowel disease, small molecule drugs

## Abstract

For a significant proportion of patients with inflammatory bowel disease (IBD), primary non-response and secondary loss of response to treatment remain significant issues. Anti-tumor necrosis factor therapies have been licensed for use in IBD. Other disease-related pathways have been targeted as well, including the interleukin 12/23 axis and lymphocyte tracking. However, the need for parenteral administration and the associated costs of dispensing and monitoring all biologics remain a burden on healthcare systems and patients. Janus kinase inhibitors are small-molecule drugs that can be administered orally and are relatively inexpensive, thus offering an additional option for treating IBD. They have been shown to be effective in patients with ulcerative colitis (UC), but they are less effective in those with Crohn’s disease (CD). Nonetheless, given the immune-system-based mechanism of these drugs, their safety profile remains a cause for concern. This article provides an overview of Janus kinase (JAK) inhibitors and new trends in the treatment of IBD.

## 1. Introduction 

Inflammatory bowel disease (IBD), including ulcerative colitis (UC) and Crohn’s disease (CD), is caused by chronic inflammation of the gastrointestinal tract [1]. Targeted biological therapies have significantly changed the management of IBD and other immune-mediated inflammatory conditions by significantly improving outcomes. Infliximab, a tumor necrosis factor (TNF)-alphareceptor blocker, was the first monoclonal antibody approved for use for the treatment of IBD in 1998. It was followed by three other anti-TNF molecules, adalimumab, golimumab, and certolizumab. Since then, other pathways have been targeted using ustekinumab and vedolizumab, which target the interleukin (IL) 12/23 axis and the lymphocyte tracking pathway, respectively. However, as these drugs cannot be administered orally, the costs associated with their delivery and monitoring are a burden for both the healthcare system and patients [2]. Therefore, a small molecule drug called the Janus kinase (JAK) pathway inhibitor, which reinforces these shortcomings, was approved for IBD treatment. 

The JAK family comprises four intracellular tyrosine kinases, JAK1, JAK2, JAK3, and tyrosine kinase 2 (TYK2), and seven transcriptional activators, which include signal transducers and intracellular transcription factors (STATs). The binding of these factors activates the JAK-STAT pathway via different cytokine receptors and leads to changes in the levels of immune mediators, such as interferons and interleukins [3]. Among the latter, IL-6 and IL-12 and IL- 23 are important drivers of disease activity in IBD [4,5]. Specifically, IL-6 is activated by JAK1, JAK2, and TYK2 via the STAT3 pathway, IL-23 via JAK2 and the STAT3 pathway, and TYK2, via the STAT4 pathway [6]. In psoriasis, rheumatoid arthritis, and other immune-mediated inflammatory conditions, blocking the JAK-STAT signaling pathway has proven to be effective in the treatment of these diseases. In the past few years, considerable interest has been devoted to the clinical development of JAK inhibitors for IBD, as the pathways involved in its pathogenesis are similar [7]. This new drug is currently being actively studied, and tofacitinib is a representative study; however, drugs other than tofacitinib are currently under active development. There is a need to review these newly developed drugs. Therefore, we wrote this article to provide an overview of JAK inhibitors and show the new trends.

An online search was performed in PubMed/MEDLINE in June 2021 to identify articles reporting JAK inhibitors for IBD treatment. The searched keywords used were “inflammatory bowel disease”, “IBD”, “ulcerative colitis”, “UC”, “Crohn’s disease”, “CD”, “small molecule”, “janus kinase inhibitor”, and “JAK inhibitor”. The reference list of the selected articles was manually investigated to confirm relevant studies. This list includes not only published studies but also e-pub or online ahead of print. Articles that cannot be interpreted because they are not written in English are excluded. The authors checked and reviewed the manuscripts of all selected studies. The relevant or comprehensive studies were finally included in our descriptive review article.

## 2. Tofacitinib in UC

Tofacitinib is an oral JAK inhibitor [8] that, in 2012, was approved for the treatment of moderate to severe active rheumatoid arthritis and in 2017 for the treatment of active psoriatic arthritis. In 2018, the European Medicines Agency approved the use of tofacitinib for UC [7]. 

In 2017, Sandborn et al. conducted three randomized, double-blind, placebo-controlled, phase 3 trials to evaluate the efficacy of tofacitinib in the treatment of UC [9]. Their OCTAVE Induction 1 and 2 trials included 598 and 541 patients with moderate to severe active UC unresponsive to conventional or TNF antagonist therapy. The patients were randomly assigned to receive either induction therapy, consisting of tofacitinib 10 mg twice daily, or placebo for 8 weeks. The primary endpoint was remission (total Mayo score ≤ 2, with no subscore > 1, and a rectal bleeding subscore of 0) at week 8. In the OCTAVE Induction 1 trial, at week 8, remission occurred in 18.5% of patients in the tofacitinib group and in 8.2% of those in the placebo group. In the OCTAVE Induction 2 trial, remission occurred in 16.6% and 3.6% of the patients, respectively. In both trials, the rates of overall infection and severe infection were higher in the tofacitinib group than in the placebo group. 

Sands et al. also published the results of the OCTAVE Sustain trial, which enrolled 593 patients with a clinical response to induction therapy, defined as a reduction in the total Mayo score of at least 3 and a ≥ 30% decrease in the rectal bleeding subscore to at least 1, or in the absolute rectal bleeding subscore to 0 or 1. These patients were randomized to receive maintenance therapy with tofacitinib (5 or 10 mg twice daily) or placebo for 52 weeks. The primary endpoint was remission at 52 weeks. The results showed that, at 52 weeks, remission was achieved in 34% of patients in the 5 mg group and 41% of those in the 10 mg group, compared to 11% in the placebo group.

In 2020, Sands et al. concluded OCTAVE Open, a long-term extension, open-label study of the OCTAVE trials that included patients already in remission on 10 mg tofacitinib twice daily; efficacy endpoints (clinical response, mucosal healing (Mayo subscore of 0 or 1) and remission) were assessed at baseline and 2, 12, and 24 months after the conclusion of OCTAVE Open. In most patients, remission was maintained following the de-escalation of tofacitinib therapy (10 mg twice daily) after 52 weeks [10]. Specifically, a clinical response was maintained in 84% of patients and remission in 75% after 52 weeks of treatment. The authors also analyzed the effects of dose escalation to tofacitinib 10 mg twice daily in patients who had lost response to tofacitinib 5 mg twice daily as maintenance therapy. A clinical response was recovered in 65% after 12 months, and 49% of patients had disease remission.

In 2021, Sandborn et al. assessed the safety and efficacy of tofacitinib 5 and 10 mg twice daily in patients who had previously failed to respond to TNF inhibitors [11]. Efficacy was assessed from the phase 3 OCTAVE Induction 1 and 2 studies of 1139 patients, the phase 3 OCTAVE Sustain maintenance study of 593 patients, and the open-label, long-term extension OCTAVE Open study with a dose-escalation subgroup of 59 patients who had received tofacitinib 5 mg twice daily in OCTAVE Sustain, and those who had received tofacitinib 10 mg twice daily in OCTAVE Open. Among all of these patients, 541 patients responded to TNF inhibitors, and 583 did not. This study demonstrated that tofacitinib was effective and safe even in patients with disease that was previously unresponsive to TNF inhibitor treatment. 

Data from the clinical use of tofacitinib in patients with UC have been reported in several retrospective studies. In 2019, Weisshof et al. conducted a retrospective observational study of the use of tofacitinib in IBD patients [12]. Those who medically tolerated previous drugs were treated orally with 5 or 10 mg tofacitinib twice daily. Clinical and adverse events were evaluated at 8, 26, and 52 weeks. Remission was defined as the complete disappearance of clinical symptoms, and endoscopic improvement as outcomes defined by a decrease in the Mayo subscore. At least 8 weeks of treatment with tofacitinib were completed by 58 patients (93% patients with failed anti-TNF). At that time, 21 patients (36%) had a clinical response, and 19 (33%) had clinical remission. Steroid-free remission at 8 weeks was achieved in 15 patients (26%), in 21% of 48 patients followed for 26 weeks, and in 27% of the 26 patients followed for 12 months. The study showed that tofacitinib induced a clinical response in 69% of patients with moderate to severe anti-TNF-resistant IBD, including 27% who were in steroid-free clinical remission by 1 year of treatment. Tofacitinib is thus an effective therapeutic option for patients with severe anti-TNF-resistant IBD.

In 2020, Honap et al. published the results of a retrospective observational cohort study performed at four centers in the UK and consisting of 134 UC patients treated with tofacitinib. The patients were enrolled from October 2018 to October 2019 and, initially, orally administered the standard tofacitinib induction dose of 10 mg twice daily; after at least 8 weeks, the dose was reduced to 5 mg twice daily [13]. In this group, 83% of the patients had previously received at least one biologic. Clinical response was defined as a reduction in the simple clinical colitis activity index (SCCAI) or the partial Mayo score (PMS) ≥ 3, and clinical remission as a SCCAI ≤ 2 or a PMS ≤ 1. The results showed that 74% of patients responded to tofacitinib at week 8, with steroid-free remission occurring in 44% of patients at week 26. Only 23% of patients who continued tofacitinib in the primary non-response setting were in steroid-free remission at week 26. Previous exposure to biologics did not affect either the response or the remission rate. However, dose escalation restored the clinical response in about half of the patients in whom it had been lost. 

In 2021, Berinstein et al. performed a retrospective case-controlled study to evaluate the efficacy of tofacitinib induction in biologically experienced patients hospitalized for acute severe UC requiring intravenous corticosteroids [14]. Forty patients receiving tofacitinib were matched 1:3 with controls (n = 113). The endpoint was colectomy within 90 days. This study showed that tofacitinib was protective against colectomy at 90 days compared to the matched controls. When stratifying according to treatment dose, 10 mg three times daily was protective, while 10 mg twice daily was not significantly protective. The rates of complications and steroid dependence were similar between the tofacitinib and control groups. The author concluded that tofacitinib with concomitant IV corticosteroids may be an effective induction strategy in biologic-experienced patients hospitalized with acute severe UC. 

In 2021, Straatmijer et al. published a retrospective cohort study that included 36 UC patients who received tofacitinib [15]. The median disease duration was 7 (3–14) years; 89% of the patients had previously failed to respond to anti-TNF treatment and 42% to vedolizumab treatment. The primary outcome also included endoscopic improvement and steroid-free clinical remission at week 52. Both endpoints were documented in 8/36 patients (22%) at 16 weeks, in 6/35 patients (17%) at 36 weeks, and in 12/31 patients (39%) at 52 weeks. The corresponding combined clinical and endoscopic response rates were 15/36 (42%), 12/35 (34%), and 15/31 (48%), and the biochemical remission rates were 11/33 (33%), 10/32 (31%), and 10/29 (34%). Patients who failed to respond to anti-TNF therapy were less likely to discontinue tofacitinib earlier than patients who had been previously anti-TNF therapy naive. Overall, the study showed that in 39% of patients with refractory UC, steroid-free clinical remission with endoscopic improvement was achieved after 1 year.

## 3. Tofacitinib in CD

In 2017, Panés et al. conducted two randomized, placebo-controlled, multicenter, phase 2b studies that evaluated the efficacy of tofacitinib as induction and maintenance therapy for CD [16]. In both, 280 adult patients with moderate to severe CD and an inadequate response to or intolerance of previously administered immunomodulators (TNF inhibitors, corticosteroids) were randomized to receive induction therapy for 8 weeks with placebo or tofacitinib 5 or 10 mg twice daily. Those who achieved clinical remission (Crohn’s disease activity index (CDAI) < 150) were rerandomized to maintenance therapy with placebo or tofacitinib 5 or 10 mg twice daily for 26 weeks. The primary endpoint was the clinical response-100 (decrease in the CDAI ≥ 100 from the induction study baseline) or clinical remission at week 26. At week 8, clinical remission was achieved in 43.5% of the patients treated with 5 mg tofacitinib twice daily and in 43.0% of those treated with 10 mg twice daily, in contrast to 36.7% in the placebo group. The differences were not significant. Although in patients treated with either dose of tofacitinib, the mean decrease in C-reactive protein (CRP) was higher than in the placebo group, there was no correlation with the mean changes in fecal calprotectin at week 8. In the maintenance study, 180 patients were rerandomized. The numbers of patients with a clinical response (decrease in the CDAI ≥ 100 from baseline) or clinical remission (CDAI < 150) were not significantly different in the tofacitinib 5 mg group (40%), the tofacitinib 10 mg group (56%), and the control group (38%). However, levels of CRP and fecal calprotectin were significantly lower in the tofacitinib 10 mg group than in the placebo group. The authors concluded that primary efficacy endpoints were not significantly different in the treated vs. the placebo group, although there was evidence of a mild therapeutic effect. 

In 2020, Fenster et al. conducted a retrospective cohort study to examine the real-world efficacy and safety of the off-label use of tofacitinib in patients with CD or unclassified IBD (IBD-U) [17]. Seventy-six patients with CD and IBD-U, 98.7% of whom had previously been treated with biologic therapy and 48.7% of whom had failed at least two biologic therapies, were followed for a median of 7.6 months. During induction therapy (75.0%) and maintenance therapy (65.3%), the dose of tofacitinib in most patients was 10 mg twice daily. The primary outcome was a clinical response (> 50% reduction in symptoms) at 8 and/or 16 weeks. Secondary outcomes were corticosteroid-free response/remission, clinical remission (no IBD symptoms), and endoscopic remission (ulcer resolution). Of the 73 patients for whom data were recorded for weeks 8 and 16, 46.6% had a clinical response, 39.7% had a corticosteroid-free clinical response, 15.1% had a clinical remission, and 13.7% had a corticosteroid-free clinical remission. At the last documented assessment (n = 75), a clinical response was determined in 42.7% (20% in clinical remission), and no or a lost response in 57.3%. Univariate comparisons of the baseline characteristics of the patients based on the clinical response at 8/16 weeks did not show significant differences in disease location, whereas male sex, body mass index, and baseline hemoglobin level were significant factors. Male sex was associated with an increased odds of a clinical response (adjusted odds ratio, 5.4) and a corticosteroid-free clinical response (adjusted odds ratio, 4.2). In patients with baseline ulceration, endoscopic remission occurred in 44% of patients starting tofacitinib treatment. Treatment outcomes were not statistically different according to the number of failed biologics prior to the initiation of tofacitinib therapy. Tofacitinib was discontinued in 4.5% of patients, most frequently due to no response (30.3%) or a loss of response (15.8%). The study showed that tofacitinib was effective in achieving a clinical response in a subset of patients with CD and IBD-U refractory to previous biologic therapies. No new significant safety issues were reported with the use of tofacitinib in these patients. 

## 4. Other Jak Inhibitors

### 4.1. Upadacitinib

Upadacitinib is an oral JAK1-selective inhibitor with a 74-fold selectivity for JAK1 over JAK2. It has a half-life of 4 h and is metabolized in the liver (80%) and eliminated by renal excretion (20%) [7]. In 2020, Sandborn et al. conducted a multicenter, double-blind, phase 2b study of 250 adults with moderate to severe active UC in whom treatment with corticosteroids, immunosuppressants, and/or biologic therapies resulted in an inappropriate response, a loss of response, or intolerance [18]. The enrolled patients were randomly assigned to placebo or induction therapy with upadacitinib (7.5, 15, 30, or 45 mg, sustained release) once daily for 8 weeks. The primary endpoint was clinical remission according to the adapted Mayo score at week 8 (stool frequency subscore of 1, rectal bleeding subscore of 0, and endoscopic subscore of 1 based on a central reading). At week 8, clinical remission was achieved in 8.5%, 14.3%, 13.5%, and 19.6% of patients receiving upadacitinib at 7.5, 15, 30, and 45 mg, respectively, but in none of the patients in the placebo group. Endoscopic improvement (defined as an endoscopic subscore of 1) at week 8 was achieved in 14.9% (7.5 mg), 30.6% (15 mg), 26.9% (30 mg), and 35.7% (45 mg) of the treated patients compared with 2.2% in the placebo group. The study showed that 8 weeks of treatment with upadacitinib was more effective than placebo in inducing remission in patients with moderate to severe active UC.

The authors also conducted a double-blind, phase II trial that included adult patients with moderate to severe CD and an inadequate response to or intolerance of immunosuppressive agents or anti-TNF inhibitor [19]. The patients were randomly assigned to the placebo group or to one of the following treatment groups: 3, 6, 12, or 24 mg upadacitinib twice daily or 24 mg upadacitinib once daily. They were assessed by ileocoloscopy at week 12 or 16 of the induction period. Patients completing week 16 were rerandomized to 36 weeks of maintenance therapy with upadacitinib. The primary outcome was clinical remission (average daily stool frequency score of 1.5 and abdominal pain score of 1.0) at week 16 and endoscopic remission (simple endoscopic score for CD of 4 and a 2-point reduction from baseline, with no subscore > 1) at week 12 or 16, determined using the multiple comparison procedure, modeling, and the Cochran-Mantel-Haenszel test, with a 2-sided level of 10%. Among the 220 patients enrolled in the study, clinical remission was achieved in 13% of those treated with 3 mg, 27% of those treated with 6 mg, 11% of those treated with 12 mg, 22% of those treated with 24 mg twice daily, 14% of those treated with 24 mg once daily, and 11% of those who had received the placebo. Endoscopic remission was achieved in 10%, 8%, 8%, 22%, and 14% of patients treated with upadacitinib but in none of the patients in the placebo group. Endoscopic remission increased with an increasing dose during the induction period. The authors concluded that efficacy was maintained for most endpoints through week 52 during the induction period. In a phase II trial in CD patients, upadacitinib induced endoscopic remission in a significant proportion of patients compared to placebo. The benefit/risk profile of upadacitinib supports its further development for the treatment of CD. 

### 4.2. Filgotinib

Filgotinib is an oral JAK1 selective inhibitor. Its half-life of 6 h for the parent compound and 23 h for the active metabolite allows once-daily dosing [7]. In 2021, Feagan et al. assessed a phase 2b/3, double-blind, randomized, placebo-controlled trial including two induction studies and one maintenance study, conducted in 341 study centers in 40 countries for filgotinib treatment of ulcerative colitis [20]. Patients were randomly assigned to receive filgotinib 100 mg, filgotinib 200 mg, or placebo, and the patients entered the maintenance study. The primary outcome was clinical remission (Mayo endoscopic subscore of 0 or 1, rectal bleeding subscore of 0, and at least a 1-point decrease in stool frequency from induction baseline for a subscore of 0 or 1) at week 10 and week 58. Some patients discontinued treatment but the proportion of patients who achieved clinical remission at week 10 was larger in the group treated with 200 mg of filgotinib than in the placebo group (induction study A: 26.1% vs. 15.3%, induction study B: 11.5% vs. 4.2%). Clinical remission at week 58 was achieved in 37.2% of patients receiving 200 mg of filgotinib compared to 11.2% in the placebo group. There was no significant difference in clinical response between filgotinib 100 mg and placebo at week 10, but the difference at week 58 was significant (23.8% vs. 13.5%). The incidence of severe adverse events and adverse events of interest was similar between treatment groups. The authors found that filgotinib 200 mg was well tolerated and effective in inducing and maintaining clinical remission in patients with moderately to severely active UC.

In 2017, Vermeire et al. conducted a randomized, double-blind, placebo-controlled phase II study that recruited patients from 52 centers in nine European countries [21]. Eligible patients ranged in age from 18 to 75 years and had a history of ileal, colonic, or ileocolonic CD (evaluated by colonoscopy and supported by histology) for 3 months or more prior to screening and a CDAI between 220 and 450 during the screening period. Enrolled patients were randomized (3:1) to receive filgotinib 200 mg once daily or placebo for 10 weeks. An interactive web-based response system was used to stratify patients according to prior anti-TNF exposure, CRP concentration at screening (≤10 mg/L or >10 mg/L), and oral corticosteroid use at baseline. The primary endpoint was clinical remission (CDAI < 150) at week 10. After 10 weeks, the patients were assigned to filgotinib 100 mg once daily, filgotinib 200 mg once daily, or placebo, depending on their responses, for an observation period lasting an additional 10 weeks. Between February 3, 2014, and July 10, 2015, 174 patients with active CD as evaluated by central read endoscopy were enrolled (130 in the filgotinib 200 mg group and 44 in the placebo group). In the intention-to-treat population, 60 of 128 patients (47%) treated with filgotinib 200 mg achieved clinical remission at week 10, compared to 10 of 44 patients (23%) treated with placebo (*p* = 0.0077). In the pooled analysis of all filgotinib and placebo exposures for 20 weeks, serious treatment-emergent adverse events developed in 14 of 152 (9%) patients treated with filgotinib and 3 of 67 patients treated with placebo. The authors concluded that filgotinib therapy could induce clinical remission in CD patients and that the safety profile was acceptable.

### 4.3. Peficitinib

Peficitinib, an oral pan-JAK inhibitor with moderate selectivity for JAK3, was approved in Japan in March 2019 for the treatment of rheumatoid arthritis in patients with an inadequate response to standard therapy. Peficitinib is metabolized in the liver, with ~57% of the dose excreted in the feces and 37% in the urine [7]. The elimination half-life of the drug is 2.8–13 h.

The safety and efficacy of pepicitinib were studied in a phase 2b dose range trial that included 219 adult patients with moderate to severe UC who were randomized to receive placebo or peficitinib 25, 75, or 150 mg or peficitinib 75 mg twice daily [22]. Although the dose response at 8 weeks was not statistically significant, a clinical response, mucosal healing, and remission were achieved in patients receiving pepicitinib > 75 mg once daily. Clinical improvement was accompanied by improvement according to the IBD questionnaire and the normalization of inflammatory biomarkers.

## 5. Safety Data

Because the immunosuppressive effects of JAK inhibition are broad, much attention has been paid to the safety profile of these drugs. Integrated safety analysis of clinical studies of tofacitinib in 1157 UC patients who were treated for up to 4.4 years, revealed that tofacitinib was globally well tolerated [23]. Safety abnormalities, such as infection, venous thrombosis, malignancy, and hyperlipidemia, were found to occur more frequently compared to placebo, but several studies concluded that IBD patients treated with JAK inhibitors had a significantly higher overall risk of side effects.

### 5.1. Infections

In the OCTAVE Induction 1 and 2 trials, severe infections occurred in six patients (1.3%) and in one (0.2%) patient in the tofacitinib 10 mg group, but in none of the patients in the corresponding placebo group. In the OCTAVE Sustain trial, severe infections occurred in two patients (1.0%) in the 5 mg tofacitinib group, in one patient (0.5%) in the 10 mg tofacitinib group, and in two patients (1.0%) in the placebo group. In the OCTAVE Induction 1 and 2 trials, herpes zoster infection occurred in three patients (0.6%) and two patients (0.5%) in the tofacitinib 10 mg group, but in only one patient and in none in the respective placebo group. Moreover, in the OCTAVE Sustain trial, herpes zoster infection occurred in three patients (1.5%) in the 5 mg tofacitinib group, in ten patients (5.1%) in the 10 mg tofacitinib group, and in one patient (0.5%) in the placebo group. The herpes zoster infections involved one dermatome or two adjacent dermatomes and did not lead to serious adverse events or drug discontinuation. In the OCTAVE Induction 2 trial, one patient in the 10 mg tofacitinib group had cytomegalovirus colitis. Tuberculosis was not reported in any of the three trials [9]. In the OCTAVE OPEN trial, serious infection was reported in one case, opportunistic infection other than herpes zoster was not reported, and herpes zoster was reported in 10 out of 123 cases [10]. In OCTAVE Sustain with prior tumor necrosis factor inhibitor failure status, serious infection was reported in one case, opportunistic infection other than herpes zoster was not reported, and herpes zoster was reported in 7 out of 57 cases [11]. 

In addition, from a systematic review in 2020, the association between tofacitinib use and infection was analyzed [24]. Serious infections were evaluated in 51 studies of 42,646 patients treated with JAK inhibitors. The global incidence rate was 3.36 per 100 patient-years, and the incidence rate among patients exposed to the comparison drug 2.01. The relative risk (RR) was 1.03; when only placebo-controlled studies were considered, the RR was 1. Herpes zoster infection was evaluated in 44 studies that included 48,093 patients exposed to JAK inhibitors. The incidence rate in 23 studies involving 9572 patients was 2.11 per 100 patient-years (rate in patients exposed to control: 1.23 per 100 patient-years). The RR of herpes zoster infection was significantly higher in patients receiving JAK inhibitors (1.57) than in controls. In an analysis of placebo-controlled studies, the RR was significant (1.72).

Pulmonary cryptococcosis, histoplasmosis, and cytomegalovirus hepatitis were included as non-herpes zoster opportunistic infections in the Global UC trial [25]. Severe non-herpes-zoster infections included two cases of anal abscesses, four of appendicitis, and two of C. difficile infection. In the CD induction studies with tofacitinib, one patient in the tofacitinib 5 mg group developed gastroenteritis and another patient an abdominal abscess; in the tofacitinib 10 mg group, one patient developed a perianal abscess and another influenza pneumonia. In the maintenance study, in the tofacitinib 5 mg twice daily group, one patient had C. difficile colitis, septic shock, and C. difficile infection and two patients had a perianal abscess; in the tofacitinib 10 mg twice daily group, there was one case of influenza pneumonia infection and one of perianal abscess. No opportunistic infections were reported. In the rheumatoid arthritis pooled trial, the risk of tuberculosis with tofacitinib varied with the background risk of the population: regions with a low to moderate tuberculosis incidence had only a small number of cases. Nevertheless, patients should be evaluated for latent and active tuberculosis prior to receiving JAK inhibitor therapy, and tuberculosis drugs should be started in those with latent tuberculosis [7]. Currently, there are many reports of infection, among which the risk of herpes zoster infection was reported the most, and some serious infections were reported. Therefore, it is considered beneficial to pre-administer the herpes zoster vaccine to the high-risk groups to prevent infection.

### 5.2. Venous Thrombotic Events

A large amount of data is available concerning the risk of deep vein thrombosis and pulmonary embolism in patients with IBD receiving tofacitinib, including a pooled analysis of 1157 patients with UC treated with tofacitinib who were enrolled in three induction studies, one maintenance trial, and one ongoing long-term extension study. In both the OCTAVE induction and maintenance (OCTAVE Sustain) studies, deep vein thrombosis (DVT)/pulmonary embolism (PE) events occurred in two placebo-treated patients. In the overall cohort, DVT/PE events occurred in five patients (one DVT and four PE), all of them in patients receiving tofacitinib 10 mg twice daily. Two patients had discontinued tofacitinib 4–5 days prior to the event, although one of them later resumed treatment with tofacitinib. All patients received a predominant dose of tofacitinib 10 mg twice daily during the entire UC clinical program [26]. To date, there have been no reports of a large number of venous thrombotic events, but it is thought that long-term data analysis will be necessary in the future.

### 5.3. Malignancy

In the overall tofacitinib trials in UC patients, during the OCTAVE Open, 11 patients had malignancies, excluding those with non-melanoma skin cancer (NMSC). These consisted of one case each of cervical cancer, hepatovascular sarcoma, cholangiocarcinoma, cutaneous leiomyosarcoma, Epstein-Barr virus-associated lymphoma, renal cell carcinoma, essential thrombocythemia, acute myeloid leukemia, colon adenocarcinoma, lung cancer, and breast cancer. A total of 8 of the 11 patients had been previously treated with TNF inhibitors and all had prior treatment with thiopurine [23]. No pattern was observed among the malignancy types. The patient with cholangiocarcinoma developed peritoneal metastasis, reported as a secondary case. Three patients died (those with hepatic angiosarcoma, cholangiocarcinoma, and acute myeloid leukemia). The incidence rate of malignancy other than NMSC, including all 11 patients who had an event, was 0.7 in the overall cohort. Another 11 patients had NMSC, six of whom had a tumor history. Four patients had basal cell carcinoma, five had squamous cell carcinoma, and two had both. Ten of the patients had previously been treated with thiopurine and ten with a TNF inhibitor. In the tofacitinib induction trials for patients with CD, one patient in the tofacitinib 10 mg twice daily treatment group developed breast cancer. No malignancies were reported in the maintenance therapy arm of the same study [16].

### 5.4. Hyperlipidemia 

Among UC patients in the tofacitinib trials, there was an increase in lipid parameters, but there were no significant changes in other laboratory parameters [23].

### 5.5. Major Adverse Cardiovascular Events 

In the overall tofacitinib trials for patients with UC, the OR of major adverse cardiovascular events (MACE) was 0.2 (95% confidence interval, 0.1–0.6), based on four patients with events [23], including one case each of hemorrhagic stroke, aortic dissection, acute coronary syndrome, and myocardial infarction. The patient with aortic dissection died. The patient with the hemorrhagic stroke event permanently discontinued tofacitinib; the patients with myocardial infarction and acute coronary syndrome temporarily discontinued tofacitinib therapy, which led to the resolution of both pathologies. Pre-existing cardiovascular risk factors were identified in the medical history of the patients with acute coronary syndrome, myocardial infarction, and hemorrhagic stroke.

### 5.6. Gastrointestinal Perforations

In the total tofacitinib trial in UC patients, the incidence rate of gastrointestinal tract perforation was 0.2, based on three patients with an event [23]. In all three patients, the perforations were considered severe adverse events. In OCTAVE Induction 1, one patient who received tofacitinib 10 mg twice daily had a perforation in the descending colon. The causes of perforation included a background of active UC inflammation, concomitant use of corticosteroids, and recent endoscopy. In the OCTAVE Open, gastrointestinal tract perforation occurred in one patient taking concomitant non-steroidal anti-inflammatory drugs, who developed appendicitis. Another patient had a sigmoid colon perforation. The background of the perforations were: at the site of Epstein-Barr viral lymphoma, a history of recent oral corticosteroids use, and recent endoscopy.

## 6. Conclusions

For many years, CD and UC have been treated mainly with corticosteroids, aminosalicylates, immunosuppressants, and TNF inhibitors. However, the recent development of small-molecule drugs and biologics has improved the treatment and thus the prognosis of IBD patients. JAK inhibitors usually can be administered orally and exert fewer adverse effects on the immune response. Therefore, the use of this drug enhances the convenience of the user. Tofacitinib was approved for patients with UC, and its efficacy has been good regardless of previous TNF inhibitor use (Table 1). However, tofacitinib has been less effective in patients with CD (Table 2). The therapeutic potential of other JAK inhibitors in the treatment of IBD has also been explored. For all of these drugs, safety remains a cause for concern and rare, albeit serious side effects, have been reported; minor or no side effects have also been reported (Table 3). Among the adverse events related to the use of JAK inhibitors is that of herpes zoster infection, but none of these cases were serious. However, as these drugs are relatively new, their long-term risks remain to be determined in future studies.

In the treatment of IBD, the use of anti-TNF agents had superior effects compared to conventional treatment. However, despite the use of biologics in many patients, problems, such as loss of response and development of drug tolerance, are appearing. Since JAK inhibitors are small molecules, they have little autoantibody formation compared to macromolecular proteins, so the possibility of response loss is expected to be relatively low, and rapid therapeutic effects are expected in patients with acute severe UC. IBD is an autoimmune disease that requires long-term treatment. Therefore, stability and continuity of drug effect are very important in treatment choice. JAK inhibitors, such as tofacitinib, open up a new horizon for small molecule drugs in IBD treatment strategies that now result in biologics. In the future, more comprehensive and head-to-head trials comparing various JAK inhibitors with existing biologics are needed. Based on the studies currently in progress and scheduled to be conducted in the future, it is expected that a new era of JAK inhibitors in the treatment of IBD will come.

## Figures and Tables

**Table 1:** JAK Inhibitors for UC treatment.

Type of JAK Inhibitor	Target	Study and Duration	Patents	Trial End Points	Treatment Phase	Treatment Groups	Results
**Tofacitinib**	Pan-JAK inhibitor	Sandborn et al. (2012) [27] 8 weeks	194	Remission on week 8	Induction	Tofacitinib 0.5 mg twice daily Tofacitinib 3 mg twice daily Tofacitinib 10 mg twice daily Tofacitinib 15 mg twice daily	The clinical response to tofacitinib was 0.5 (32%), 3 (48%), 10 (61%), and 15 mg (78%) compared with 42% of the placebo group. The clinical remission rate, defined by Mayo score ≤2, was placebo group 10% and 0.5 (13%), 3 (33%), 10 (48%), and 15 mg (41%), respectively.
		Sandborn et al. (OCTAVE In-duction 1) (2017) [9] 8 weeks	598	Remission on week 8	Induction	Placebo Tofacitinib 10 mg twice daily	Remission at 8 weeks occurred in 18.5% of the patients in the tofacitinib group versus 8.2% in the placebo group.
		Sandborn et al. (OCTAVE In-duction 2) (2017) [9] 8 weeks	541	Remission on week 8	Induction	Placebo Tofacitinib 10 mg twice daily	Remission occurred in 16.6% of the patients in the tofacitinib group versus 3.6% in the placebo group.
		Sandborn et al. (OCTAVE Sustain) (2017) [9] 52 weeks	593	Remission on week 52	Maintenance	Placebo Tofacitinib 5 mg twice daily Tofacitinib 10 mg twice daily	Remission at 52 weeks occurred in 34.3% of the patients in the 5 mg tofacitinib group and 40.6% in the 10 mg tofacitinib group versus 11.1% in the placebo group.
		Patel et al. (2019) [28] 8 weeks	123	Remission on week 8	Induction	Tofacitinib 10 mg twice daily	At 8 weeks, 60.8% had a clinical response, and 13.5% had clinical remission. At 16 weeks, 55.4% had a clinical response and 48.6% had clinical remission, and 64.9% showed mucosal healing findings on endoscopy. There was no difference in clinical response between those who had not previously used biologics and those who had used anti-TNF agents.
		Weisshof et al. (2019) [12] 52 weeks	58	Remission, endoscopic improvement, failure response	Induction and maintenance	Tofacitinib 5–10 mg twice daily	During the 8 week treatment course, 36% of patients had a clinical response and 33% of patients had clinical remission. Steroid-free remission at 8 weeks was 26% and 21% at 26 weeks. After 12 months at follow up, 27% of patients had clinical, steroid-free remission.
		Clark et al. (2019) [29] >4 weeks	24	SCCAI, Mayo endoscopic score decrease at week 4–20	Induction and mainte nance	Tofacitinib 5–10 mg twice daily	Clinical disease activity after 4–20 weeks of tofacitinib improved in both the UC (SCCAI decreased from 7.18 ± 2.97 to 4.53 ± 3.44, *p* = 0.009) and the CD population (HBI decreased from 10.86 ± 7.51 to 7.6 ± 5.73, *p* = 0.02). Endoscopic disease activity after 8–53 weeks of tofacitinib trended down in both UC (Mayo endoscopic score decreased from 2.21 ± 1.18 to 1.25 ± 1.49, *p* = 0.36) and CD populations (SES-CD decreased from 20.67 ± 13.31 to 11.81 ± 12.06, *p* = 0.07).
		Kolar et al. (2019) [30] 8 weeks	24	Mayo endoscopic subscore decrease at week 8	Induction	Tofacitinib 10 mg twice daily	Total Mayo decreased in responders from mean 5.9 ± 3.5 to 1.1 ± 1.3 (*p* = 0.01), while in nonresponders it changed from 8.0 ± 2.5 to 8.9 ± 2.1 (*p* = 0.86). Endoscopic subscore decreased from 2.0 ± 1.0 to 0.6 ± 0.7 (*p* = 0.02) in responders but remained stationary in nonresponders.
		Honap et al. (2019) [31] 8 weeks	16	SCCAI decrease at week 8	Induction	Tofacitinib	Median baseline SCCAI (n = 8) fell from 8 (range 2–14) to 3 (1–5) after four weeks and remained stable at eight weeks. Median baseline faecal calprotectin (n = 5) fell from 364 (131–645) to 95 (30–289).
		Sands et al. (2020) [10] 12 month	57	Clinical response, mucosal healing, remission at month 2, month 12	Maintenance (dose escalation)	Tofacitinib 10 mg twice daily	57.9% (33/57) and 64.9% (37/57) of patients recaptured clinical response and 35.1% (20/57) and 49.1% (28/57) were in remission, at months 2 and 12, respectively.
		Sands et al. (2020) [10] 12 month	66	Clinical response, mucosal healing, remission at month 2, month 12	Maintenance (dose de-escalation)	Tofacitinib 5 mg twice daily	92.4% (61/66) and 84.1% (53/63) of patients maintained clinical response and 80.3% (53/66) and 74.6% (47/63) maintained remission, at months 2 and 12, respectively.
		Mehiri et al. (2020) [32] 48 weeks	38	Survival without colectomy and without tofacitinib discontinuation, steroid-free clinical remission at 8 weeks	Induction and maintenance	Tofacitinib 10 mg twice daily for 3 months, then tofacitinib 5 mg or 10 mg twice daily	Survival without colectomy was 77% at week 24 and 70% at week 48. Survival without treatment discontinuation was 70% at week 24. Steroid-free clinical remission was observed in 13 (34%) patients at week 48.
		Biemans et al. (2020) [33] 24 weeks	123	Endoscopic, clinical, biologic remission at 24 weeks	Induction and maintenance	Tofacitinib 10 mg twice daily for 8 weeks, then tofacitinib 5 mg twice daily	Corticosteroid-free clinical, biochemical, and combined corticosteroid-free clinical and biochemical remission rate at week 24 was 29%, 25%, and 19%, respectively. Endoscopic remission (Mayo = 0) was achieved in 21% of patients at week 12.
		Berinstein et al. (2021) [14] 90 days	113	Colectomy within 90 days	Induction	Tofacitinib 10 mg twice daily	Tofacitinib with concomitant IV corticosteroids may be an effective induction strategy in biologic-experienced patients hospitalized with acute severe UC.
		Straatmijer et al. (2021) [15] 52 weeks	36	Combined with SCCAI ≤2 and Mayo score ≤1 at 52 weeks.	Maintenance	Tofacitinib 5–10 mg twice daily	Corresponding combined clinical and endoscopic response rates were 42%, 34%, and 48%, and biochemical remission rates were 33%, 31%, and 34%.
		Sandborn et al. (2021) [11] Long term	1124		Induction, maintenance	Placebo Tofacitinib 5–10 mg twice daily	Treatment effects were higher for tofacitinib 10 mg or tofacitinib 5 mg twice daily than placebo.
**Upadacitinib**	JAK1 selective inhibitor	Sandborn et al. (2018) [34] 8 weeks	250	Clinical remission	Induction	Placebo Upadacitinib 7.5 mg once daily Upadacitinib 15 mg once daily	There was significant improvement of individuals in the treatment group through endoscopic, histologic, and clinical remission at week 8, particularly at doses of 15 mg or higher, compared with placebo.
		Sandborn et al. (2020) [18] 8 weeks	250	Clinical remission according to the adapted Mayo score at week 8	Induction	Placebo upadacitinib 7.5 mg once daily Upadacitinib 15 mg once daily Upadacitinib 30 mg once daily Upadacitinib 45 mg once daily	At week 8, 8.5%, 14.3%, 13.5%, and 19.6% of patients receiving 7.5, 15, 30, or 45 mg upadacitinib, respectively, and achieved clinical remission compared with none of the patients receiving placebo. Endoscopic improvement at week 8, defined as endoscopic subscore of 1, was achieved in 14.9%, 30.6%, 26.9%, and 35.7% of patients receiving upadacitinib 7.5, 15, 30, or 45 mg, respectively, compared with 2.2% receiving placebo.
**Filgotinib**	JAK1 selective inhibitor (highly selective JAK1)	SELECTION trial (2020) [35] 10 weeks	1348	Clinical remission at 10 weeks	Induction	Placebo Filgotinib 200 mg once daily Filgotinib 100 mg once daily	At 10 weeks, compared with the control group (15.3%), the treatment group had a high clinical remission rate of 26.1%. In the biologic-naïve patient group, the Mayo Clinic Score remission rate was 24.5%, the endoscopic remission rate was 12.2%, and the histological remission rate was 35.1%. Control groups were 12.4%, 3.6%, and 16.1%. In the biologic-experienced patient group, the clinical remission rate was 11.5% and 4.2% in the control group.
		SELECTION trial (2020) [35] 58 weeks	558	Clinical remission at 58 weeks	Maintenance	Placebo Filgotinib 200 mg once daily	In the group treated with filgotinib 200 mg, the clinical remission rate was 18.1%, the MCS remission rate was 34.7%, the endoscopic remission rate was 15.6%, and the histological remission rate was 38.2%. In the control group, it was 5.1%, 9.2%, 6.1%, and 13.3%, respectively. Additionally, a significantly higher proportion of patients treated with filgotinib 200 mg achieved six-month corticosteroid-free clinical remission at week 58 compared with placebo (27.2% vs. 6.4%).
		Feagan et al. (2021) [20] 58 weeks	689	Clinical remission at 58 weeks	Induction	Filgotinib 100 mg once daily Filgotinib 200 mg once daily Placebo	In induction study A, the clinical remission rates were 26.1% in the treatment group and 15.3% in the control group, and in induction study B, 11.5% in the treatment group and 4.2% in the control group. At week 58, 37.2% of patients given filgotinib 200 mg had clinical remission versus 11.2% in the respective control group. Clinical remission was not significantly different between filgotinib 100 mg and placebo at week 10, but at 58 weeks, the treatment group was 23.8%, which was significantly different from the control group 13.5%.
**Peficitinib**	Pan-JAK inhibitor	Sandborn et al. (2018) [22] 8 weeks	219	Clinical remission at 8 weeks	Induction	Placebo Peficitinib 25 mg once daily Peficitinib 75 mg once daily Peficitinib 150 mg once daily Peficitinib 75 mg twice daily	No statistically relevant difference between treatment and placebo groups.

JAK, Janus kinase inhibitor; UC, ulcerative colitis; SCCAI, simple clinical colitis activity index; CD, Crohn’s disease; HBI, Harvey–Bradshaw index; SES-CD, simple endoscopic score for Crohn’s disease.

**Table 2:** JAK Inhibitors for CD treatment.

Type of JAK Inhibitor	Target	Study and Duration	Patents Number	Trial End Points	Treatment Phase	Treatment Groups	Results
**Tofacitinib**	Pan-JAK inhibitor	Sandborn et al. (2014) [36] 4 weeks	139	Clinical response at 4 weeks	Induction	Placebo Tofacitinib 1 mg twice daily Tofacitinib 5 mg twice daily Tofacitinib 15 mg twice daily	Clinical response was observed in 36%, 58%, and 46% of patients given the 1, 5, and 15 mg doses of tofacitinib, compared with 47% of patients given placebo. Clinical remission was observed in 31%, 24%, and 14% of patients given the 1, 5, and 15 mg doses of tofacitinib, compared with 21% of patients given placebo.
		Panés et al. (2017) [16] 8 weeks	280	Clinical response-100 or clinical remission at 8 weeks	Induction	Placebo Tofacitinib 5 mg twice daily Tofacitinib 10 mg twice daily	The clinical remission rate was 43.5% in the tofacitinib 5 mg group and 43% in the 10 mg group.In the control group, it was 36.7%.
		Panes et al. (2017) [16] 26 weeks	180	Clinical response-100 or clinical remission at 26 weeks	Maintenance	Placebo Tofacitinib 5 mg twice daily Tofacitinib 10 mg twice daily	The clinical remission rate was 55.8% in the tofacitinib 10 mg group and 39.5% in the 5 mg group.In the control group, it was 38.1%.
		Panés et al. (2019) [37] 48 weeks	150	Mainteined remission at 48 weeks	Maintenance	Tofacitinib 5 mg twice daily (patients in remission) Tofacitinib 10 mg twice daily (patients not in clinical remission)	Crohn’s disease worsening was the most frequent adverse event for tofacitinib 5 (33.9%) and 10 mg (19.3%).
		Clark- et al. (2019) [29] >4 weeks	24	HBI score, endoscopic disease activity declined	Induction and maintenance	Tofacitinib 5–10 mg twice daily	Significant improvement in HBI score (10.86 ± 7.51 to 7.60 ± 5.73). The endoscopic disease activity declined (from 20.67 ± 13.31 to 11.81 ± 12.06), C-reactive protein declined (from 24.83 ± 24.68 to 17.92 ± 22.63).
		Weisshof et al. (2019) [12] 52 weeks	4		Induction and maintenance	Tofacitinib 5–10 mg twice daily	One patient interrupted treatment because of unsatisfactory response, and the other three patients showed clinical response using tofacitinib.
**Upadacitinib**	JAK1 selective inhibitor	Sandborn et al. (2017) [38] 16 weeks	220	Endoscopic and clinical remissions at 16 weeks	Induction	Placebo Upadacitinib 3 mg twice daily Upadacitinib 6 mg twice daily Upadacitinib 12 mg twice daily Upadacitinib 24 mg twice daily Upadacitinib 24 mg once daily	Compared with placebo, more patients achieved clinical response with 6 and 24 mg twice daily, and endoscopic response with upadacitinib doses ≥6 mg twice daily at week 16.
		Sandborn et al. (2018) [38] 16 weeks	180	Endoscopic and clinical remissions at 16 weeks	Maintenance	Upadacitinib 3 mg twice daily Upadacitinib 12 mg twice daily Upadacitinib 24 mg once daily	Dose-dependent enhancements in endoscopic and clinical remissions and inflammation markers were observed with upadacitinib treatment.
**Filgotinib**	JAK1 selective inhibitor	Vermeire et al. (2017) [21] 10 weeks	173	Clinical remission at 10 weeks	Induction	Placebo Filgotinib 200 mg once daily	A total of 47% of patients treated with filgotinib 200 mg achieved clinical remission at week 10 versus 23% of patients treated with placebo.

JAK, Janus kinase inhibitor; CD, Crohn’s disease; HBI, Harvey–Bradshaw index.

**Table 3:** Adverse events reported in JAK inhibitors.

Adverse Event	Type of JAK Inhibitor	Incidence Rate	Notes
Serious Infections	Tofacitinib	1.37%, Sandborn et al. (2012) [22] 2.66%, Panes et al. (2019) [37] 2.85%, Sandborn et al. (2019) [23]	Serious infection rates increased depending on whether or not the body weight was greater than 90 kg (HR, 2.3) [23].
	Filgotinib	2.63%, Vermeire et al. (2017) [21]	
Herpes zoster	Tofacitinib	1.17%, Panes et al. 2017 [16] 3.29%, Sandborn et al. 2017 [9] 2.0%, Panes at al. 2019 [37] 5.62%, Sandborn et al. 2019 [23]	Older age (every 10 years; HR, 1.6), prior tumor necrosis factor inhibitor failure (HR, 1.9), and nonwhite race (white vs. all other races; HR, 0.6) were significant risk factors for herpes zoster.The IR for herpes zoster in Asian patients was 6.5 versus 3.5 in white patients.
	Upadacitinib	0.54%, Sandborn et al. 2018 [34]	
	Filgotinib	0.66%, Vermeire et al. 2017 [21]	
Thrombotic Events	Tofacitinib	0.43%, Sandborn et al. 2019 [23]	PE in the setting of cholangiocarcinoma metastasized to the peritoneum.
Non-melanoma skin cancer	Tofacitinib	0.76%, Sandborn et al. 2017 [9] 0.66%, Panes et al. 2019 [37] 0.95%, Sandborn et al. 2019 [23]	Age (every 10 years; HR, 2.2) and prior tumor necrosis factor inhibitor failure (yes vs. no; HR, 11.3) were significant risk factors.
	Upadacitinib	0.54%, Sandborn et al. 2018 [34]	
Other malignancy	Tofacitinib	0.58%, Panes et al. 2017 [16] 0.0%, Sandborn et al. 2017 [9] 0.0%, Panes et al. 2019 [37] 0.95%, Samdborn et al. 2019 [23]	
Major Adverse Cardiovascular Events	Tofacitinib	0.51%, Sandborn et al. 2017 [9] 0.0%, Panes et al. 2019 [37] 0.34%, Samdborn et al. 2019 [23]	Pre-existing cardiovascular risk factors were identified for the acute coronary syndrome, myocardial infarction, and hemorrhagic stroke events.

JAK, Janus kinase inhibitor; HR, hazard ratio; IR, incident rate; PE, pulmonary embolism.

## References

[1] Pippis E.J., Yacyshyn B.R. (2020). Clinical and Mechanistic Characteristics of Current JAK Inhibitors in IBD. Inflam. Bowel. Dis..

[2] Harris C., Cummings J.R.F. (2021). JAK1 inhibition and inflammatory bowel disease. Rheumatology.

[3] Coskun M., Salem M., Pedersen J., Nielsen O.H. (2013). Involvement of JAK/STAT signaling in the pathogenesis of inflammatory bowel disease. Pharmacol. Res..

[4] Mudter J., Neurath M.F. (2007). Il-6 signaling in inflammatory bowel disease: Pathophysiological role and clinical relevance. Inflamm. Bowel. Dis..

[5] Uhlig H.H., McKenzie B.S., Hue S., Thompson C., Joyce-Shaikh B., Stepankova R., Robinson N., Buonocore S., Tlaskalova-Hogenova H., Cua D.J. (2006). Differential activity of IL-12 and IL-23 in mucosal and systemic innate immune pathology. Immunity.

[6] Galien R. (2016). Janus kinases in inflammatory bowel disease: Four kinases for multiple purposes. Pharmacol. Rep..

[7] Garrido I., Lopes S., Macedo G. (2021). Hit the Road JAK! The Role of New Oral Treatment in Inflammatory Bowel Disease. Inflam. Bowel. Dis..

[8] Burmester G.R., Nash P., Sands B.E., Papp K., Stockert L., Jones T.V., Tan H., Madsen A., Valdez H., Cohen S.B. (2021). Adverse Events of Special Interest in Clinical Trials of Rheumatoid Arthritis, Psoriatic Arthritis, Ulcerative Colitis and Psoriasis with 37 066 Patient-Years of Tofacitinib Exposure. RMD. Open.

[9] Sandborn W.J., Su C., Sands B.E., D’Haens G.R., Vermeire S., Schreiber S., Danese S., Feagan B.G., Reinisch W., Niezychowski W. (2017). OCTAVE Induction 1, OCTAVE Induction 2, and OCTAVE Sustain Investigators Tofacitinib as Induction and Maintenance Therapy for Ulcerative Colitis. N. Engl. J. Med..

[10] Sands B.E., Armuzzi A., Marshall J.K., Lindsay J.O., Sandborn W.J., Danese S., Panés J., Bressler B., Colombel J.F., Lawendy N. (2020). Efficacy and Safety of Tofacitinib Dose De-Escalation and Dose Escalation for Patients with Ulcerative Colitis: Results from OCTAVE Open. Aliment. Pharmacol. Ther..

[11] Sandborn W.J., Peyrin-Biroulet L., Sharara A.I., Su C., Modesto I., Mundayat R., Gunay L.M., Salese L., Sands B.E. (2021). Efficacy and Safety of Tofacitinib in Ulcerative Colitis Based on Prior Tumor Necrosis Factor Inhibitor Failure Status. Clin. Gastroenterol. Hepatol..

[12] Weisshof R., Aharoni Golan M., Sossenheimer P.H., El Jurdi K., Ollech J.E., Pekow J., Cohen R.D., Sakuraba A., Dalal S., Rubin D.T. (2019). Real-World Experience with Tofacitinib in IBD at a Tertiary Center. Dig. Dis. Sci..

[13] Honap S., Chee D., Chapman T.P., Patel M., Kent A.J., Ray S., Sharma E., Kennedy J., Cripps S., Walsh A. (2020). LEO [London, Exeter, Oxford] IBD Research Consortium Real-World Effectiveness of Tofacitinib for Moderate to Severe Ulcerative Colitis: A Multicentre UK Experience. J. Crohns Colitis.

[14] Berinstein J.A., Sheehan J.L., Dias M., Berinstein E.M., Steiner C.A., Johnson L.A., Regal R.E., Allen J.I., Cushing K.C., Stidham R.W. (2021). Tofacitinib for Biologic-Experienced Hospitalized Patients with Acute Severe Ulcerative Colitis: A Retrospective Case-Control Study. Clin. Gastroenterol. Hepatol..

[15] Straatmijer T., van Gennep S., Duijvestein M., Ponsioen C.I.J., Gecse K.B., D’Haens G.R., Löwenberg M. (2021). Real-World Clinical and Endoscopic Outcomes After One Year Tofacitinib Treatment in Ulcerative Colitis. Eur. J. Gastroenterol. Hepatol..

[16] Panés J., Sandborn W.J., Schreiber S., Sands B.E., Vermeire S., D’Haens G., Panaccione R., Higgins P.D.R., Colombel J.F., Feagan B.G. (2017). Tofacitinib for Induction and Maintenance Therapy of Crohn’s Disease: Results of Two phase IIb Randomized Placebo-Controlled Trials. Gut.

[17] Fenster M., Alayo Q.A., Khatiwada A., Wang W., Dimopoulos C., Gutierrez A., Ciorba M.A., Christophi G.P., Hirten R.P., Ha C. (2021). Real-World Effectiveness and Safety of Tofacitinib in Crohn’s Disease and IBD-U: A Multicenter Study from the TROPIC Consortium. Clin. Gastroenterol. Hepatol..

[18] Sandborn W.J., Ghosh S., Panes J., Schreiber S., D’Haens G., Tanida S., Siffledeen J., Enejosa J., Zhou W., Othman A.A. (2020). Efficacy of Upadacitinib in a Randomized Trial of Patients with Active Ulcerative Colitis. Gastroenterology.

[19] Sandborn W.J., Feagan B.G., Loftus E.V., Peyrin-Biroulet L., Van Assche G., D’Haens G., Schreiber S., Colombel J.F., Lewis J.D., Ghosh S. (2020). Efficacy and Safety of Upadacitinib in a Randomized Trial of Patients with Crohn’s Disease. Gastroenterology.

[20] Feagan B.G., Danese S., Loftus E.V., Vermeire S., Schreiber S., Ritter T., Fogel R., Mehta R., Nijhawan S., Kempinski R. (2021). Filgotinib as induction and maintenance therapy for ulcerative colitis (SELECTION): A phase 2b/3 double-blind, randomised, placebo-controlled trial. Lancet.

[21] Vermeire S., Schreiber S., Petryka R., Kuehbacher T., Hebuterne X., Roblin X., Klopocka M., Goldis A., Wisniewska-Jarosinska M., Baranovsky A. (2017). Clinical Remission in Patients with Moderate-to-Severe Crohn’s Disease Treated with Filgotinib (the Fitzroy Study): Results from a phase 2, Double-Blind, Randomized, Placebo-Controlled Trial. Lancet.

[22] Sands B.E., Sandborn W.J., Feagan B.G., Lichtenstein G.R., Zhang H., Strauss R., Szapary P., Johanns J., Panes J., Vermeire S. (2018). Peficitinib-UC Study Group Peficitinib, an Oral Janus Kinase Inhibitor, in Moderate-to-Severe Ulcerative Colitis: Results from a Randomized, phase 2 Study. J. Crohns Colitis.

[23] Sandborn W.J., Panés J., D’Haens G.R., Sands B.E., Su C., Moscariello M., Jones T., Pedersen R., Friedman G.S., Lawendy N. (2019). Safety of Tofacitinib for Treatment of Ulcerative Colitis, Based on 4.4 Years of Data from Global Clinical Trials. Clin. Gastroenterol. Hepatol..

[24] Olivera P.A., Lasa J.S., Bonovas S., Danese S., Peyrin-Biroulet L. (2020). Safety of Janus Kinase Inhibitors in Patients with Inflammatory Bowel Diseases or Other Immune-Mediated Diseases: A Systematic Review and Meta-Analysis. Gastroenterology.

[25] Winthrop K.L., Melmed G.Y., Vermeire S., Long M.D., Chan G., Pedersen R.D., Lawendy N., Thorpe A.J., Nduaka C.I., Su C. (2018). Herpes Zoster Infection in Patients with Ulcerative Colitis Receiving Tofacitinib. Inflam. Bowel. Dis..

[26] Sandborn W.J., Panés J., Sands B.E., Reinisch W., Su C., Lawendy N., Koram N., Fan H., Jones T.V., Modesto I. (2019). Venous Thromboembolic Events in the Tofacitinib Ulcerative Colitis Clinical Development Programme. Aliment. Pharmacol. Ther..

[27] Sandborn W.J., Ghosh S., Panes J., Vranic I., Su C., Rousell S., Niezychowski W. (2012). Study A3921063 Investigators Tofacitinib, an Oral Janus Kinase Inhibitor, in Active Ulcerative Colitis. N. Engl. J. Med..

[28] Patel A., Fenster M., Bader G., Dimopoulos C., Deepak P., Ungaro R.C., Ciorba M.A., Yarur A.J., Hirten R., Christophi G.P. (2019). Real-World Effectiveness of Tofacitinib in Ulcerative Colitis; a Multi-Center Study. Gastroenterology.

[29] Clark-Snustad K., Singla A., Lee S.D. (2019). Tofacitinib Improves Clinical Disease Activity in a Real-World Population of Patients with Moderate-Severe Ulcerative Colitis and Crohn’s Disease. Gastroenterology.

[30] Kolar M., Lukas M., Malickova K., Prochazkova L., Bortlik M., Duricova D., Hruba V., Machkova N., Mitrova K., Vasatko M. (2020). P368 Tofacitinib Induction Efficiency and Intracellular Cytokine Dynamics in Ulcerative Colitis: Results from Clinical Practice. J. Crohns Colitis.

[31] Honap S., Sharma E., Ray S., Cunningham G., Tamilarasan A., Mawdsley J., Anderson S., Sanderson J., Irving P. (2019). Early Real–Real World’ Experience with Tofacitinib for Moderate to Severe Ulcerative Colitis. Gut.

[32] Lair-Mehiri L., Stefanescu C., Vaysse T., Laharie D., Roblin X., Rosa I., Treton X., Abitbol V., Amiot A., Bouguen G. (2020). Real-World Evidence of Tofacitinib Effectiveness and Safety in Patients with Refractory Ulcerative Colitis. Dig. Liver. Dis..

[33] Biemans V.B.C., Sleutjes J.A.M., de Vries A.C., Bodelier A.G.L., Dijkstra G., Oldenburg B., Löwenberg M., van Bodegraven A.A., van der Meulen-de Jong A.E., de Boer N.K.H. (2020). Dutch Initiative on Crohn and Colitis (ICC) Tofacitinib for Ulcerative Colitis: Results of the Prospective Dutch Initiative on Crohn and Colitis (ICC) Registry. Aliment. Pharmacol. Ther..

[34] Sandborn W., Ghosh S., Panés J., Schreiber S., D’Haens G.R., Tanida S., Siffledeen J., Enejosa J., Zhou W., Othman A.A. (2018). Efficacy and Safety of Upadacitinib as an Induction Therapy for Patients with Moderate-to-Severely Active Ulcerative Colitis: Data from the Phase 2 B Study U-ACHIEVE. United. Eur. Gastroenterol. J..

[35] Gilead Sciences Galapagos N.V. A phase 2b/3 Trial Showed the Efficacy of Filgotinib for the Induction and Maintenance of Remission in Moderately and Severely Active Ulcerative Colitis. https://www.businesswire.com/news/home/20201012005470/en/.

[36] Sandborn W.J., Ghosh S., Panes J., Vranic I., Wang W., Niezychowski W. (2014). Study A3921043 Investigators A Phase 2 Study of Tofacitinib, an Oral Janus Kinase Inhibitor, in Patients with Crohn’s Disease. Clin. Gastroenterol. Hepatol..

[37] Panés J., D’Haens G.R., Higgins P.D.R., Mele L., Moscariello M., Chan G., Wang W., Niezychowski W., Su C., Maller E. (2019). Long-Term Safety and Tolerability of Oral Tofacitinib in Patients with Crohn’s Disease: Results from a phase 2, Open-Label, 48-Week Extension Study. Aliment. Pharmacol. Ther..

[38] Sandborn W.J., Feagan B.G., Panes J., D’Haens G.R., Colombel J.F., Zhou Q., Huang B., Enejosa J.V., Pangan A.L., Lacerda A.P. (2017). Safety and Efficacy of ABT-494 (Upadacitinib), an Oral JAK1 Inhibitor, as Induction Therapy in Patients with Crohn’s Disease: Results from Celest. Gastroenterology.

